# Assessment of burnout among primary teachers in confinement during the COVID-19 period in Morocco: case of the Kenitra

**DOI:** 10.11604/pamj.supp.2020.35.2.24345

**Published:** 2020-06-25

**Authors:** Abdeslam Amri, Zakaria Abidli, Mohamed Elhamzaoui, Mounir Bouzaboul, Ziri Rabea, Ahmed Omar Touhami Ahami

**Affiliations:** 1 Cognitive Behavioral Neuroscience and Applied Nutrition Team, Laboratory Nutrition-Health and Environment, Department of Biology, Faculty of Sciences, University IBN TOFAIL, Kenitra, Morocco; 2 Ministry of National Education, Professional Training, Higher Education and Scientific Research, Kenitra, Morocco

**Keywords:** Burnout, teachers, primary, confinement, COVID-19, Morocco

## Abstract

**Introduction:**

Confinement, because of the COVID-19 pandemic, could have problems on the mental health of the population. Teachers responsible for giving distance courses during this period could be psychologically stressed. The aim is to assess the magnitude results of burnout and associated factors among primary school teachers in Kenitra in Morocco during this confinement period.

**Methods:**

This is a cross-sectional study, which was conducted during the two months of April and May 2020. Burnout was evaluated by the Maslach Burnout Inventory MBI with 16 items, specific to the context of Moroccan teachers and the factors of stress were assessed using a questionnaire developed by the research team. We used the Chi-square test to determine the association between two qualitative variables anda logistic regression for an overall statistical analysis.

**Results:**

The average age was 38.6 ± 9.9 years. The MBI revealed that 68 teachers (54%) were victims of burnout, of which 47 (38%) had a low level; 15 (12%) had a moderate level and six (5%) had a severe burnout. Logistic regression analysis has shown that the risk factors for burnout during this confinement period are: the use and development of skills in new information and communication technologies (p<0,05); work/family conflict (p<0,05); social support (p<0,05); and the workload related to distance education (p≤0,05).

**Conclusion:**

In light of these results, interventions aimed at promoting mental well-being teachers during and after confinement should be implemented immediately.

## Introduction

COVID-19 is an emerging infectious disease attributed to a new SARS-CoV-2 coronavirus, detected for the first time, in China, in the city of Wuhan on December 31, 2019 [1]. Only a month after its emergence, the World Health Organization (WHO) declared the COVID-19 epidemic as a public health emergency of international concern. Because of its rapid and accelerated spread, it declared it, on March 11, 2020, as a global pandemic [2]. In Morocco the first cases of COVID-19 were reported on March 2 and since March 16, classroom courses have been suspended in all educational establishments and are replaced by distance courses [3]. Discussions with many teachers in confinement directed our attention to repeat complaints from some primary school teachers about the difficulties of teaching at a distance. They mainly complain about the qualification and skills in new information and communication technologies (NICT), workload, social support, work-family role conflict, lack of equipment computer science, etc. They feel harassed by the accumulation of demands and by a drought of resources to cope. Some of them can no longer adapt, making them potential candidates for burnout that has been defined as a negative psychic experience, lived by an individual, linked to chronic emotional stress caused by work aimed at helping people [4]. A reflection on these complaints led us to take an interest in the suffering of this active population and the repercussions of these working conditions at a distance on the mental health of primary school teachers in confinement from Kenitra. In this sense, we aimed to assess burnout and determine its prevalence and its risk factors among this population.

## Methods

This cross-sectional study was conducted during April and May 2020. It concerned primary school teacher´s in confinement from Kenitra in Morocco. We assessed the socio-demographic data by a self-questionnaire and those related to work by a questionnaire developed by the research team based on the literature review [5]. The latter, examined by three experts in occupational psychology and by two arab linguists, aimed to assess the requirements of distance education during this period of confinement and the resources these teachers have to cope. This 15-item questionnaire measures four dimensions on a Likert scale from 1 to 4: work overload with 4 items; the conflict of roles between teaching at a distance and the responsibilities of the family with 4 items, the use and development of skills in new information and communication technologies (NICT) with 4 items and support social from the hierarchy, colleagues and family with 3 items. Burnout was evaluated by the Maslach Burnout Inventory MBI with 16 items, specific to the context of Moroccan teachers, validated in the laboratory of applied neurosciences, in the department of biology, at the faculty of science of Kenitra. The psychometric validation of this version has been the subject of a scientific publication accepted by a journal indexed scopus, it is in the process of being published.

This version kept the three-dimensionality of Maslach’s theoretical model and assesses the burnout syndrome through its three dimensions: emotional exhaustion (6 items), depersonalization (6 items) and sense of personal accomplishment (4 items). This arabic version showed Cronbach’s alpha index values good for emotional exhaustion (0.81) and depersonalization (0.85) and satisfactory for the feeling of personal accomplishment (0.79) [6]. The participants have filled datasheet electronically and the investigation contacted primary school teachers in Kenitra. The investigation was anonymous and the confidentiality of the information was guaranteed. The statistical analyzes were made using the SPSS version 21 statistical software. Thus, to extract the requirements felt by teachers in confinement and the resources to cope, a principal component analysis with varimax rotation was performed on the items of the questionnaire developed by the research team. The association between burnout and socio-demographic risk factors on the one hand and those linked to distance education on the other was made by the Chi-square test, the level of significance was fixed at α ≤0,05. To determine the potential risk factors for burnout in the participants, a logistic regression analysis was performed and the associations between the risk factors and the results are presented in the form of odds ratios (OR) and 95% CI.

## Results

For the 125 participants who completed the technical sheet, the average age was 38.6 ± 9.9 years, with a minimum age of 25 and a maximum age of 59. The sex ratio was 1.3 in favor of the female sex. The average professional seniority was 13.9 ± 8.9 years. 58.4% are between 25 and 40 years old; 56.8% are female; 73.6% are married and 75.2% have a professional service of less than 20 years (Table 1).

**Table 1:** Sociodemographic characteristics and factors related to distance education of primary school teachers in confinement (n=125) Kenitra, (May/April 2020)

Variable	Effective	Percentage
**Age**		
25-40	73	58.4%
41-59	52	41.6%
**Sex**		
Female	71	65.8%
Male	54	43.2%
**Marital status**		
Married	92	73.6%
Unmarried	33	6.4%
**Professional seniority**		
<20	94	75.2%
≥20	31	24.8%
**Workload**		
High	69	55.2%
Low	56	44.8%
**Work-Family conflicts**		
High	70	56%
Low	55	44%
**Use and development of ICT skills**		
Low	61	48.8%
High	64	51.2%
**Social support**		
Low	102	81.6%
High	23	18.4%

### Factorial analyzes and internal validity of measurement instruments

**Requirements and resource:** after calculating the internal consistency coefficient (Cronbach’s alpha) for the entire questionnaire, we found that it is weak and does not allow an exploratory approach to be carried out, which led us to delete two items. The deletion of items 7 and 12 made it possible to achieve a satisfactory internal consistency coefficient (Cronbach’s alpha = 0.77) and a principal component analysis (PCA) with varimax rotation was carried out (very significant Bartlett sphericity test; index Kayser-Meyer-Olkin: KMO = 0.798). This analysis made it possible to retain 13 items distributed over four main dimensions explaining 66.58% of the variance: workload (5 items), work-family conflict (3 items), use and skills development (2 items) and social support (3 items); the internal consistency coefficients were all satisfactory. 0.856 for workload, 0.884 for work-family conflict, 0.722 for use and skills development and 0.713 for social support.

**Figure 1:**
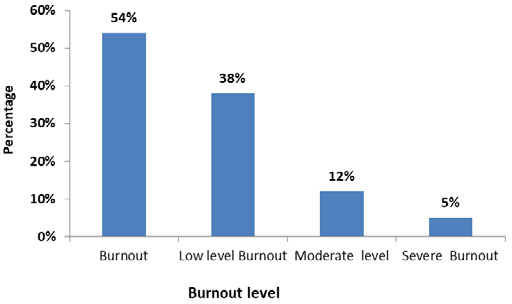
Profile of burnout syndrome among teachers in our population

**Burnout:** using the same procedure for the 16-item MBI, specific to Moroccan teachers, the PCA with varimax rotation made it possible to retain the 16 items distributed over three main dimensions, explaining 71.93% of the variance: emotional exhaustion (6 items), depersonalization (6 items) and sense of personal accomplishment (4 items). The internal consistency coefficients were all satisfactory, 0.908 for emotional exhaustion, 0.832 for depersonalization and 0.748 for sense of personal accomplishment. For the characteristics related to distance education during this confinement period, 55.23% (n = 69) perceived a heavy workload, 56% (n = 70) declared having a work-family conflict, 48.8% (n = 61) have not used and developed their ICT skills very much and 81.6% (n = 102) perceived weak support from their hierarchy, their colleagues and even their family members (Table 1). For the three dimensions of burnout, (emotional exhaustion, depersonalization and sense of personal accomplishment), average scores were respectively, 20.8 ± 8.8, 11.1 ± 9.0 and 13.1 ± 6.0. Pathologically, emotional exhaustion was high in 24.0%, depersonalization was high in 15.2% and sense of personal accomplishment was low in 39.0 % of participants (Table 2). Similarly, the MBI revealed that 68 teachers (54%) were victims of burnout, of which 47 (38%) had a low level; 15 (12%) had a moderate level and six (5%) had a severe burnout (Figure 1).

**Table 2:** Averages, standard deviations and prevalence of the dimensions of burnout among primary school teachers in confinement (n=125) Kenitra, (May/April 2020)

Variable	Averages	Standard deviations	Pathological cases	Prevalence
Emotional exhaustion	20.8	8.8	30	24%
Depersonalization	11.1	9.0	12	15.2%
Sense of personal accomplishment	13.1	6.0	49	39.2%

Concerning socio-demographic risk factors, the Chi-square test showed that gender and marital status have no significant link with burnout, while age was found to be significantly associated, participants over the age of 41 were more exhausted (p<0.01). Seniority was also significantly associated with burnout: teachers with seniority over 20 years were the most exhausted (p<0.05) (Table 3). For the risk factors linked to working at a distance, during this confinement period, the Chi-square test showed that the burnout of Kenitra primary school teachers was significantly linked to: the workload (p<0.01), the work/family conflicts (p<0.01), to the use and development of skills in new information and communication technologies (NICT) (p <0.001) and to social support from the hierarchy, colleagues and family members (p<0.001) (Table 4). Logistic regression analysis has shown that the risk factors related to distance work associated with burnout by primary teachers of the Kenitra during this confinement period are: the use and development of skills in new information and communication technologies (NICT); the distance work/family responsibility conflict; social support (hierarchy, colleagues, family) and the work load (Table 4).

**Table 3:** Association of burnout with sociodemographic and work-related risk factors among primary teachers in confinement (n=125) Kenitra, (May/April 2020)

Variable	Burnout		No Burnout		Chi-square	p-value
	Effective	Percentage	Effective	Percentage		
**Age**						
25-40	32	47%	41	71.9%	6.32	0.009**
41-59	36	53%	16	28.1%		
**Sex**						
Female	42	61.8%	29	50.9%	1.83	N.S
Male	26	38.2%	28	35.1%		
**Marital status**						
Married	55	80.9%	37	64.9%	2.02	N.S
Unmarried	13	13.1%	20	35.1%		
**Professional seniority**						
<20	45	66.2%	49	86%	5.54	0.015*
≥20	23	33.8%	8	14%		
**Workload**						
High	48	70.6%	21	36.8%	7.11	0.006**
Low	20	29.4%	36	63.2%		
**Work-Family conflicts**						
High	46	67.5%	24	42.1%	6.09	0.01**
Low	22	32.5%	33	57.9%		
**Use and development of ICT skills**						
Low	41	60.3%	20	35.1%	7.89	0.001***
High	27	39.7%	37	64.9%		
**Social support**						
Low	63	92.6%	39	68.4%	12.12	0.000***
High	5	7.4%	18	31.6%		

N.S: No significant;

* Significant at the 0.05 level (bilateral);

** significant at the 0.01 level (bilateral);

*** Significant at the 0.001 level (bilateral)

**Table 4:** Logistic regression analysis of independent predictors of burnout among primary school teachers in confinement (n=125) Kenitra, (May/April 2020)

	A	P-value	Odds-ratio	CI 95%	
Work-Family conflicts	901	0.03	1,998	1,162	5,312
Use and development of ICT skills	990	0.01	2,692	1,131	6,403
Workload	804	0.05	2,234	974	5,127
Social support	-1,221	0.02	295	099	880
Age	205	0.67	1,228	468	3,220
Professional seniority	894	0.12	2,446	780	7,665

CI: Confidence Interval

## Discussion

The period of confinement due to the spread of COVID-19 has negative effects on the psychological state of the world´s population. This has been explained by several researches. Indeed, a study done in China showed that more than 25% of the general population experienced moderate to severe levels of symptoms related to stress or anxiety in response to COVID-19 [7,8]. Similarly, an analysis and synthesis of existing evidence on the prevalence of depression, anxiety and insomnia among healthcare professionals during the COVID-19 outbreak had shown that anxiety has been assessed in 12 studies, with a pooled prevalence of 23.2% and depression in 10 studies, with a prevalence rate of 22.8% [9]. These studies are in harmony with the study carried out by the high commission for planning of Morocco, which demonstrated that for 49% of households, anxiety is the main psychological impact of confinement followed by the feeling of claustrophobia (30% of households) and the increase in phobias for 25% of households in a Moroccan population [10]. Unfortunately, these studies did not target the psychological state of teachers in confinement and according an overview on scientific publications at the level of the large Scopus and Sciencedirect databases, we noted that no study has been carried out on the mental health of Moroccan teachers. For this reason, the objective of our study was to assess burnout and determine its prevalence and associated risk factors among a sample of Moroccan primary teachers during confinement.

This cross-sectional survey included 125 respondents and revealed a high prevalence of burnout; 54.4% of all participants reported symptoms of burnout. Logistic regression showed that the burnout of primary school teachers in Kenitra, during the confinement period, was associated with workload (p<0.05) and work/family role conflict (p<0.05), these two variables are job requirements. Kenitra primary teachers in confinement therefore suffer from a workload linked to the pressure of time to accomplish their tasks remotely; to the intensity and complexity of the tasks to be carried out. They are also harassed by the responsibilities devoted to their families, especially when they coincide with the tasks of distance education. Likewise, logistic regression has shown that burnout is associated with the use and development of ICT skills (p<0.05) and social support (p<0.05). These two variables are resources available to teachers to meet requirements; participants who have little used and developed ICT skills and those who received little support from their hierarchy, their colleagues or their families were more exposed to burnout. The results found in this study are consistent with those of a meta-analysis covering one hundred and seventy-nine articles, or 186,440 subjects dealing with the associations between burnout on the one hand and the demands and resources perceived at work [11].

In this study, we tried to explain burnout through the job demands, job resources (JD-R) model of Schaufeli and Bakker (2004), which postulates the existence of two processes acting in parallel to predict the consequences of working conditions on health [12]. First, the energy process which focuses on the constraints and requirements of the job and are the ones that will cause the worker to expend energy and become exhausted. Then, the motivational process which postulates that the resources of the worker will allow him to engage in his work (Bakker and Demerouti, 2017) [13]. Regarding the burnout of Kenitra teachers in confinement, we can conclude that it is the result of an energy process, with a negative feedback on the requests via the low resources of the teachers, which could decrease, destabilize and undermine their performances, which then amplifies more the process leading to burnout.

## Conclusion

In this study, Kenitra Primary School teachers in confinement reported a high burnout rate. The protection of teachers must be an important component of public health measures to combat the consequences of the COVID-19 epidemic on both their mental and physical health. Interventions to promote the mental well-being of teachers during and after confinement must be implemented immediately and older teachers require special attention.

### What is known about this topic

- During this period of confinement, the mental health of the population has been affected;
- Burnout is very widespread among teachers around the world and the stressors associated with distance learning are factors that favor it;

### What this study adds

- Factors associated with burnout among primary school teachers in confinement were associated with the perceived levels of requirements and available resources;
- This study could offer avenues of work for management programs with a view to providing cognitive-behavioral remediation to teachers suffering from burnout during and after this period of confinement and also for a comparison with other studies;
- Inform the ministry responsible for the seriousness of the psychological state of the teachers during the confinement period in order to prepare a suitable device in the event of the appearance of the second wave, especially with regard to distance education.

## Competing interests

The authors declare no competing interests.
